# Comparison of combined compression and surgery with high ligation-endovenous laser ablation-foam sclerotherapy with compression alone for active venous leg ulcers

**DOI:** 10.1038/s41598-019-50617-y

**Published:** 2019-10-01

**Authors:** Xiaochun Liu, Guofu Zheng, Bo Ye, Weiqing Chen, Hailiang Xie, Teng Zhang

**Affiliations:** Department of Vascular and Hernial Surgery, Ganzhou People’s Hospital (The Affiliated Ganzhou hospital of Nanchang University), No. 17, Red flag avenue, Ganzhou city, Jiangxi Province 341000 PR China

**Keywords:** Peripheral vascular disease, Peripheral vascular disease, Skin manifestations, Skin manifestations

## Abstract

We aimed to assess the ulcer healing time and recurrence rates after treatment with compression therapy (CT) with or without high ligation-endovenous laser ablation-foam sclerotherapy (HL-EVLA-FS) in people with active venous leg ulcers (VLUs). A retrospective cohort study was conducted with 350 patients with active VLUs treated by compression with or without HL-EVLA-FS in our hospital from 2013 to 2017. The primary outcome was the ulcer healing time; secondary outcomes were the 12-month recurrence rates, the relationship between recurrence and venous reflux, and the complications of the two treatments. In total, 193 patients (200 limbs) underwent compression plus HL-EVLA-FS, and 157 patients (177 limbs) underwent CT alone. The ulcer healing time was shorter in the compression plus HL-EVLA-FS group than in the CT alone group (Hazard Ratio [HR] for ulcer healing, 1.845 [95% CI, 1.474–2.309], P = 0.0001). The 12-month ulcer recurrence rates were significantly reduced in the compression plus HL-EVLA-FS group (HR for ulcer recurrence, 0.418 [95% CI, 0.258–0.677], P = 0.0001). Calf perforator vein reflux (CPVR) and isolated superficial venous reflux (ISVR) were risk factors for ulcer recurrence. The combined operation with CT resulted in faster healing of VLUs, a lower ulcer recurrence rate and lower VCSS values after intervention than CT alone.

## Introduction

An active VLU is the highest clinical stage of lower limb chronic venous insufficiency (C6)^1^. Its clinical characteristics are severe symptoms, slow healing, and a proneness to recurrence, and the prevalence increases with age^2^. In addition, due to the high cost of treatment^3^, VLUs have been considered as one of the common diseases endangering public health. Sustained superficial venous hypertension caused by venous reflux is the main pathological basis for VLUs^4,5^. CT^6,7^ and various kinds of surgery (traditional surgery, ultrasound-guided foam sclerotherapy [UGFS]^8,9^, EVLA^10,11^ and radiofrequency ablation [RA]^12^) have good effects on eliminating or reducing superficial venous hypertension and promoting ulcer healing. In the Effect of Surgery and Compression on Healing and Recurrence (ESCHAR) study^13,14^, CT combined with surgery was thought to have a lower ulcer recurrence rate. However, the study found no difference in ulcer healing time between the two groups. Recently, another clinical trial (EVRA ulcer trial)^3^ concluded that CT combined with early endovenous ablation treatment could promote ulcer healing, reduce ulcer recurrence and prolong the patients’ ulcer-free time within 1 year after the intervention. The effect of compression combined use of HL-EVLA-FS is to eliminate superficial venous reflux and cure active VLUs, but it is rarely reported. We aimed to assess the ulcer healing time and recurrence rates after treatment with compression with or without HL-EVLA-FS in people with active VLUs.

## Patients and Methods

### Patients

This retrospective cohort study was approved by the medical ethics committee of Ganzhou People’s Hospital. The methods were carried out in accordance with the approved guidelines.

The study was based on a consecutive inpatient population with active VLUs and was conducted in a single centre from 2013 to 2017.

The inclusion criteria were as follows:

1. Age ≥ 18 years;
2. Active venous ulcer of the lower extremity;
3. Ultrasonographic examination indicating venous reflux;
4. Patients with VLUs who had never received compression therapy.

The exclusion criteria were as follows:

1. Leg ulcers from other causes, such as arterial ulcers, diabetic ulcers, malnutrition ulcers, and malignant ulcers, and with an ankle–brachial index < 0.8;
2. Healed ulcers (C5);
3. A history of surgery for VVs;
4. Diameter of VVs ≥ 1 cm;
5. Serious systemic diseases;
6. Patients who could not tolerate compression therapy;
7. Patients with VLUs who had chosen other therapies.

Eligible patients’ personal and clinical details, including gender, age, body mass index (BMI), history of diabetes, smoking history, course of varicose veins, ulcer site, ulcer duration, ulcer diameter, venous reflux pattern, and preoperative (Venous Clinical Severity Score)VCSS^16^, were recorded at baseline.

## Methods

### Therapy options

According to the information provided in the informed consent form in the medical record, the therapy option was based on the preference expressed by either the patient or the treating clinician. Therapy options included CT with or without HL-EVLA-FS.

Patients satisfying the inclusion criteria were fully informed about CT with or without HL-EVLA-FS and gave their written consent to undergo this specific treatment.

### Venous reflux

A time of 500 ms was recommended as the cutoff value for saphenous, deep femoral, and perforator vein incompetence, and 1 s, for femoral and popliteal vein incompetence^17^.

Venous reflux was identified as isolated superficial venous reflux (ISVR), superficial venous reflux (SVR), segmental deep venous reflux (SDVR), full-length deep venous reflux (FLDVR) and calf perforator veins reflux (CPVR) according to the location of reflux^4,18^.

### Surgical therapy

#### HL-EVLA

All the varicose veins were marked before surgery. High ligation was performed at the SFJ^19^. The trunk of the GSV was punctured at the medial malleolus, and the laser fibre was inserted into the trunk of the GSV. The 810-nm laser instrument (AngioDynamics, Germany) was activated using a pulse of 1 time/second to close the trunk of the GSV. For those patients in whom the guidewire could not be inserted upward from the ankle, such as in those with local infection or stenosis of the GSV, we chose to downwardly insert the guidewire into the GSV at the inguinal incision and retrogradely or segmentally cauterize the GSV using a laser. HL and EVLA of the SSV were performed on patients with ulcers located on the lateral malleolus. All surgical interventions were performed under spinal or general anaesthesia^19^.

#### FS

Multi-point punctures were made in the VVs of the calf. Sclerosing foam was injected into the VVs separately under non-ultrasound-guided foam sclerotherapy (N-UGFS) (foam was produced with the use of the Tessari technique at a ratio of 1 ml of 1% lauromacrogol to 4 ml of CO_2_). Approximately 4 ml of sclerosing foam was injected at each point. The total amount of lauromacrogol injected into each limb was no more than 10 ml.

### Compression therapy

CT included the early treatment of compression bandages and the post-treatment of compression hosiery^20^.

CT was administered by therapists or trained community and hospital-based nursing teams. Following cleansing of the ulcer, the wound was covered with Vaseline gauze. The limb was pressurized with a four-layer compression bandage, ensuring 40 mm Hg of compression at the ankle, the leg pressure gradually diminishing up the leg. After 3 days, the bandages were replaced by level 2 compression hosiery. The length of bed rest was 6 hours after surgery. Continuous compressive therapy was administered during the first two weeks. After 2 weeks, the patients were instructed to wear level 2 compression hosiery during the day and to remove the hosiery at night and elevate the limb.

### Follow-up protocol

Patients with active VLUs followed up monthly at outpatient visits after the intervention, and the ulcers were photographed. If clinically necessary, patients would receive more outpatient follow-up visits. The patients underwent reexamination at 1 month, 6 months and 12 months, respectively, after the intervention. The reexamination method was physical examination and duplex scanning. During the follow-up, some patients might have changed their treatment methods, such as adding surgery. Patients with ulcer healing continued to follow up for 12 months after healing. If ulcers recurred, the patient would be admitted to the hospital immediately.

### Outcomes

#### Primary outcome

The primary endpoint was time to ulcer healing. Ulcer healing time was the time from the beginning of the intervention to the time of ulcer healing. Ulcer healing was the complete epithelialization of the leg ulcer and was assessed throughout the 1-year follow-up period by the therapists or trained community and hospital-based nursing teams (at least once a month).

#### Secondary outcomes

Included the changes in VCSS at 1 month, 6 months and 12 months, the 12-month recurrence rates, ulcer recurrence correlations with venous reflux patterns and complications in the two groups. Time zero for ulcer recurrence was at ulcer healing for those with open ulcers after intervention; therefore, ulcer recurrence analyses only included patients with healed ulcers. Ulcer recurrence is the return of ulceration at the healed ulcer site, presenting as incomplete epithelium.

### Statistical analysis

The continuous variables of the two samples were described by means ± standard deviations (SDs), and their differences were compared by independent-sample *t*-tests. Categorical data are presented as percentages. We analysed results with Kaplan-Meier life tables, with a log-rank test of the two groups for time to ulcer healing and recurrence. The HR for time to ulcer healing and recurrence of the two interventions was calculated using Cox regression analysis. Multivariate Cox regression was used to analyse the relationship between ulcer recurrence and the venous reflux patterns, and the log-rank test was used to compare the risk factors for ulcer recurrence in venous reflux patterns.

All the tests were two-sided, with a significance level of 0.05, and were performed using SPSS software (ver. 22.0; IBM Corp; USA).

## Results

### General data of patients

**Figure 1:**
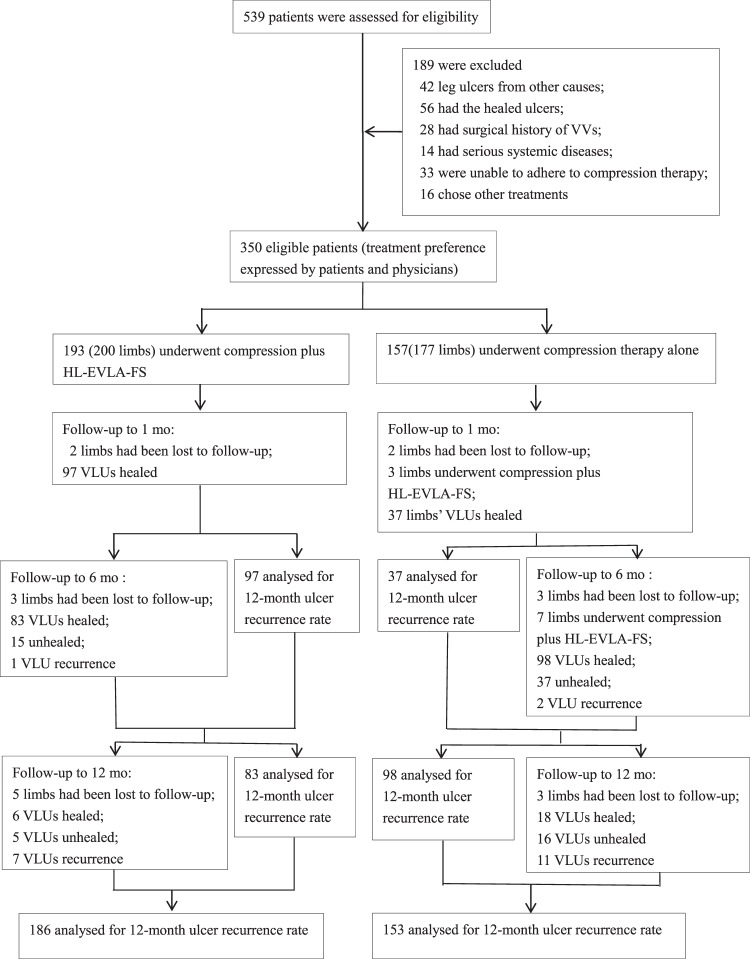
Assessment for Eligibility, groups, and Outcomes. VLUs, venous leg ulcers; VVs, varicose veins; HL-EVLA-FS, high ligation- endovenous laser ablation- foam sclerotherapy.

In total, 539 consecutive patients with a history of chronic leg ulceration were assessed for inclusion. A total of 189 patients were excluded (Fig. 1), of whom 42 had leg ulcers from other causes, 56 had healed ulcers, 28 had surgical history of VVs, 14 had serious systemic diseases, 33 were unable to adhere to CT, and 16 chose other treatments. The number of eligible cases was 350. Based on the preference expressed by either the patient or the treating clinician, 193 patients (200 limbs) underwent compression plus HL-EVLA-FS, and 157 patients (177 limbs) underwent compression therapy alone. Many of whom came from countryside. Baseline characteristics were similar in the two groups (Table 1). Factors considered to affect ulcer healing, such as gender, age, BMI, course of varicose veins, ulcer duration, ulcer diameter, venous reflux, and VCSS before intervention, did not differ between the two groups.

**Table 1:** Baseline Characteristics of the Study Patients According to Treatment Group.

Characteristic	Compression plus HL-EVLA-FS (N = 200)	Compression therapy alone (N = 177)	P-value
Gender			0.899^§^
Male	97(48.5%)	87(49.2%)	
Female	103(51.5%)	90(50.8%)	
Age	60.6 ± 10.3	62.4 ± 12.2	0.124*
Hospital stay	11.2 ± 5.3	10.8 ± 5.7	0.479*
BMI	28.4 ± 2.69	28.4 ± 2.73	0.984*
History of diabetes	7(3.5%)	6(3.4%)	0.935^§^
Smoking history	61(30.5%)	55(31.1%)	0.904^§^
Come from countryside	170(85%)	146(82.5%)	0.508^§^
Course of varicose vein	20.5 ± 12.4	22.3 ± 8.77	0.107*
Ulcer duration	25.3 ± 71.0	17.2 ± 41.3	0.169*
Ulcer diameter	3.09 ± 2.36	3.11 ± 1.72	0.920*
Ulcer site			0.626^§^
Right medial malleolus	42(21%)	35(19.8%)	
Right lateral malleolus	8(4%)	12(6.8%)	
Right foot boots area	25(12.5%)	20(11.3%)	
Left medial malleolus	81(40.5%)	63(35.6%)	
Left lateral malleolus	11(5.5%)	15(8.5%)	
Left foot boots area	33(16.5%)	32(18.1%)	
Venous reflux			
SDVR	50(25.0%)	46(26.0%)	0.826^§^
FLDVR	46(23.0%)	40(22.6%)	0.926^§^
CPV	74(37.0%)	65(36.7%)	0.956^§^
SVR	177(88.5%)	157(88.7%)	0.951^§^
ISVR	70(37.0%)	67(37.9%)	0.565^§^
The VCSS of pre-intervention	12.5 ± 3.31	12.2 ± 2.6	0.260*

*independent-sample *t*-test, ^§^Pearson Chi-Square.

HL-EVLA-FS high ligation-endovenous laser ablation-foam sclerotherapy, BMI body mass index, ISVR isolated superficial venous reflux, SVR superficial venous reflux, SDVR segmental deep venous reflux, FLDVR full-length deep venous reflux, CPVR Calf perforator veins reflux.

HL-EVLA was applied to the GSV of 200 limbs and to the SSV of 20 limbs. N-UGFS injection was performed on the VVs of 200 limbs. The average amount of lauromacrogol solution was 9.5 ± 1.0 ml/limb. Both groups received CT with 4-layer bandages for 3 days in the early stage and hosiery in the later stage. The number of limbs lost to follow-up at 1, 6 and 12 months after intervention was 2, 3 and 5, respectively, in the combined treatment group and 2, 3 and 3, respectively, in the CT alone group; in the two groups, 4 and 6 limbs, respectively, with healed ulcers were lost to follow-up 12 months after ulcer healing. The 18 and 10 patients lost to follow-up are shown as censored cases in the relevant healing and recurrence rate analyses, respectively. In addition, in the CT alone group, considering the possible poor outcomes, 3 and 7 limbs were found to have received combined treatment at 1 month and 6 months, respectively. These patients remained in the CT alone group for analysis to overcome baseline bias. At 12 months of observation after the intervention, 6 and 16 limbs remained unhealed in the combined treatment group and the CT alone group, respectively (Fig. 1).

### Primary outcome

**Figure 2:**
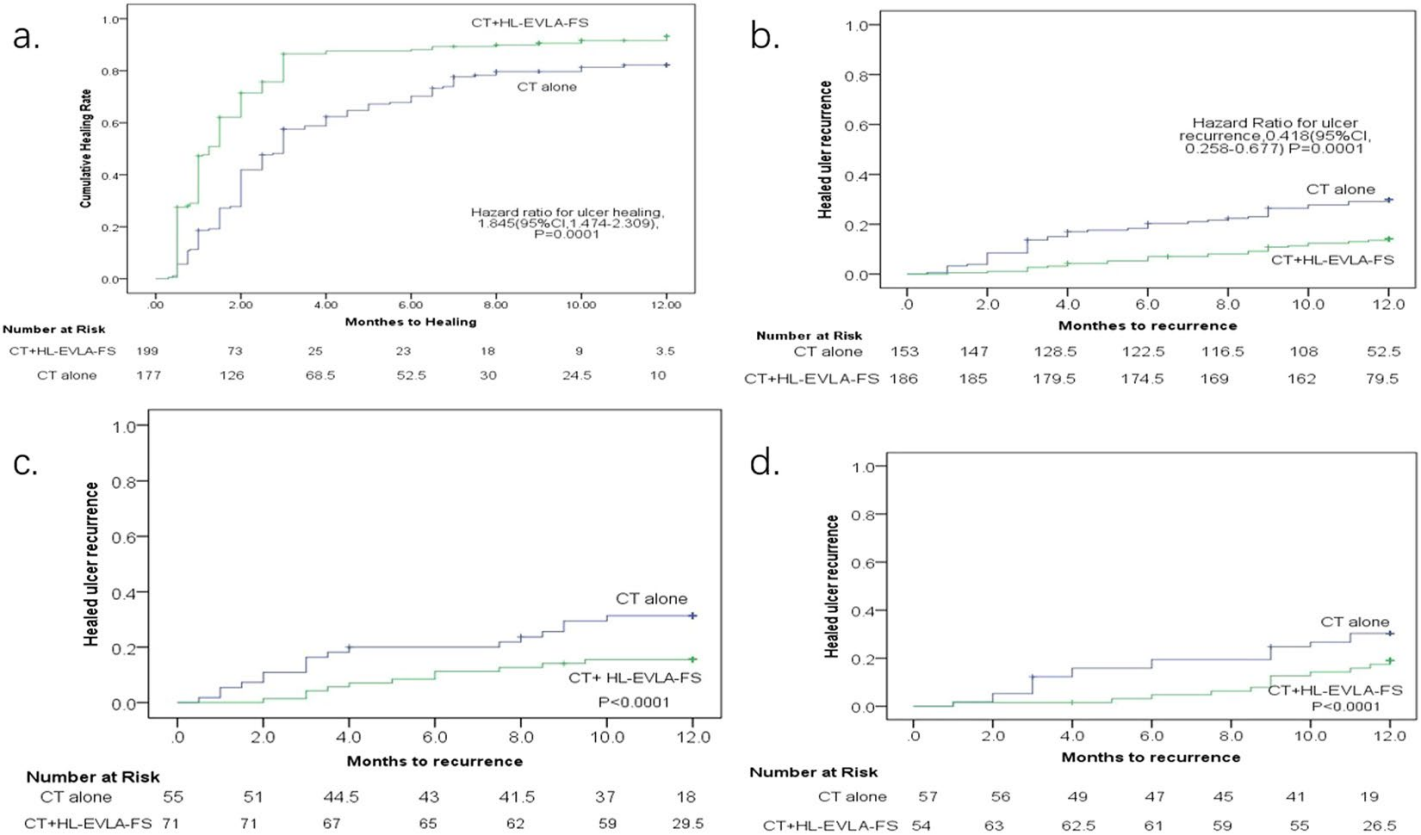
(**a**) Kaplan-Meier analysis of ulcer healing for all legs. (**b**) Kaplan-Meier analysis of ulcer12-month recurrence for all healed legs. (**c**) Kaplan-Meier analysis of ulcer recurrence by the reflux pattern of CPVR. (**d**) Kaplan-Meier analysis of ulcer recurrence by the reflux pattern of ISVR.

After 12 months of intervention, 186 limb ulcers healed in the combined treatment group with a median healing time of 1.08 months (95% CI, 1.02–1.36), while 153 limb ulcers healed in the CT alone group with a median healing time of 2.15 months (95% CI, 1.92–2.45). The ulcer healing time was shorter in the combined treatment group than in the CT alone group (HR for ulcer healing, 1.845 [95% CI, 1.474–2.309], P = 0.0001) (Fig. 2a). After adjusting for age, BMI, duration of varicose veins, ulcer duration, and ulcer diameter, the result was consistent. Ulcer healing was faster in the combined treatment group (HR for ulcer healing, 1.938 [95% CI, 1.544–2.434], P = 0.0001).

### Secondary outcomes

There were significant differences in VCSS values between the two groups at 1 month, 6 months and 1 year after intervention. The combined treatment group had lower VCSS values (Table 2).

**Table 2:** The changes in VCSS at 1 month, 6 months and 12 months after intervention.

Time	Compression plus HL-EVLA-FS (N = 200)	Compression therapy alone (N = 177)	P- value*
1 month post-intervention	9.77 ± 3.62	12.3 ± 2.73	0.000
6 months post-intervention	2.56 ± 1.92	7.60 ± 2.88	0.000
12 months post-intervention	2.17 ± 1.81	6.76 ± 2.61	0.000

*independent-sample *t*-test.

Follow-up continued for 12 months after ulcer healing. The number of ulcer recurrent limbs (in previously healed limbs) in the combined treatment group was 26 (186), while it was 45 (153) in the CT alone group. The combined treatment group had obviously lower ulcer recurrence than the CT alone group (HR for ulcer recurrence, 0.418 [95% CI, 0.258 to 0.677], P = 0.0001) (Fig. 2b).

The venous reflux modes were taken into account in the multivariate Cox regression analysis. We found that CPVR (HR for ulcer recurrence, 7.734 [95% CI, 1.513 to 39.532], P = 0.014) and ISVR (HR for ulcer recurrence, 4.070 [95% CI, 1.229 to 13.478], P = 0.0022) were risk factors for ulcer recurrence (Table 3). The log-rank test was used to compare the influence of the risk factors of CPVR (Fig. 2c) and ISVR (Fig. 2d) on ulcer recurrence in the two groups, and the results showed that the P values were both less than 0.0001.

**Table 3:** Multivariate COX regression analysis the venious reflux pattern related to the ulcer recurrence.

Variable	HR	95% CI	P-value
SDVR	2.179	0.196–24.191	0.526
FLDVR	1.661	0.212–12.994	0.629
CPVR	**7.734**	**1.513**–**39.532**	**0.014**
SVR	1.554	0.178–13.565	0.690
ISVR	**4.070**	**1.229**–**13.478**	**0.022**
SDVR and CPVR	0.333	0.079–1.413	0.136
SVR and SDVR	1.462	0.153–13.973	0.741
SVR and FLDVR	1.535	0.176–13.416	0.698
SVR and CPVR	0.351	0.078–1.578	0.172

HR hazard ratio, CI confidence interval, ISVR isolated superficial venous reflux, SVR superficial venous reflux, SDVR segmental deep venous reflux, FLDVR full-length deep venous reflux, CPVR Calf perforator veins reflux.

For complications in the combined treatment group, DVT occurred in 2 limbs (1.0%), although the thrombus disappeared at 3 months and 6 months after anticoagulation therapy and compression treatment, respectively; laser burn of the skin occurred in 6 limbs (3.0%), but the symptoms were mild and resolved within 1 week; and saphenous nerve injury occurred in 11 limbs (5.5%) and presented as skin numbness on the inner side of the calf. After 3 months, the symptoms gradually eased, and after 6 months, they disappeared; superficial phlebitis occurred in 15 limbs (7.5%), and the symptoms disappeared within 3 months after compression treatment, elevation of limbs and application of mucopolysaccharide polysulfonate cream. The common complication of CT was skin anaphylaxis to hosiery in 35 cases (9.3%). After anti-allergic treatment, all cases improved.

## Discussion

Through the retrospective cohort study, we found that combined surgery with CT had a shorter ulcer healing time, lower ulcer recurrence rate and better VCSS value improvement after intervention than CT alone for lower extremity active VLUs.

The principle of treating VLUs of lower limbs is to eliminate or reduce venous reflux, reduce superficial venous hypertension and promote ulcer healing by various methods^21^. According to the extent of the lesion and for better treatment effects, combined treatment modalities have been constantly appearing^22^. Combined treatments, such as EVLA-FS^23,24^, HL-FS^25^, and HL-EVLA^26^, have been introduced successively. Comprehensive treatments can draw on their respective strengths to achieve the best effect, especially when treating severe or recurrent VVs^24,25,27^. However, the combined treatment of active VLUs with HL-EVLA-FS plus CT has rarely been reported.

The combined treatment of HL-EVLA-FS is feasible for the treatment of VLUs because it conforms to the treatment principle of VVs. First, the reflux of the saphenous veins is blocked by high ligation of these vessels and their branches. Second, the saphenous veins are closed with EVLA, which blocks the veins themselves. Third, FS is used to close the VVs, which addresses the offending vessels, eliminates the reflux target vessels of the PVs and narrows them. FS also directly acts on the endothelial cells of the PVs, causing their occlusion. Finally, compression can damage the endothelium by sclerosing agents adhering to each other and preventing the VVs from being re-opened by blood flow. All these strategies help to eliminate venous reflux, reduce superficial venous compression and promote ulcer healing. A variety of treatments have been used to eliminate total superficial vein reflux in treating venous ulcers, and good results have been achieved^28^.

In an early clinical study (the ESCHAR study) according to the venous reflux characteristics of limbs with VLUs, patients were randomized into two groups, comparing CT alone and compression plus surgical treatment (disconnecting the saphenofemoral junction, stripping of the saphenous vein and avulsing calf varicosity) of the superficial vein under general anaesthesia or local anaesthesia; the researchers concluded that combined treatments could not accelerate ulcer healing but could reduce ulcer recurrence^13,14^. This finding was different from our conclusion and might be related to the surgical mode of intervention. However, the clinical study concluded that patterns of venous reflux, such as ISVR, were associated with ulcer recurrence. In our study, Cox regression analysis of venous reflux patterns also showed that ulcer recurrence was associated with ISVR and CPVR.

Recently, another clinical trial (the EVRA Current Controlled Trials number, ISRCTN02335796.) evaluating the role of early endovenous treatment of superficial venous reflux as an adjunct to compression therapy in patients with venous leg ulcers was performed^15^. In the trial, ablation methods included EVLA or RA, UGFS, etc. The authors concluded that CT combined with early endovenous ablation treatment could promote ulcer healing, reduce ulcer recurrence and prolong the patients’ ulcer-free time within 1 year after the intervention. Our conclusion of this retrospective analysis was similar to that of this trial. However, there was no difference in VCSS values between the two groups during their follow-up.

Compression therapy of the VLUs is usually done with bandages or hosiery, but it is still unclear which is the best option^29^. A randomized controlled study on the bandages and hosiery for the treatment of VLUs indicated that double hosiery could provide 40 mm Hg pressure at the ankle and was an effective alternative to the four-layer bandage. The extra benefit of two-layer hosiery was to reduce ulcer recurrence and cost. However, compared with the bandages, the treatment changes with the hosiery were higher, but not all people were suitable for the hosiery^7^. The bandage must be used by a specially trained physician or nurse to achieve the right pressure. After patient activity, the bandage tends to be easily shifted. Therefore, every 2–3 days, the bandage needs to be adjusted. The use of hosiery is convenient. The patient, family members, or community nurse can accomplish appropriate maintenance. Our choice is to use bandages in the early treatment of VLUs for three days in hospital and then use hosiery after three days.

Moreover, we directly injected foam sclerosing agent into VVs without ultrasound or catheter guidance with no resultant pulmonary embolism. To achieve ulcer healing, Bush *et al*.^30^ used a percutaneous approach of non-ultrasound guided injection of foam sclerosing agent directly into the VVs around the ulcer to close the VVs and PVs surrounding the ulcer. During our early surgery, we found that the injection of foam sclerosing agent into the superficial veins could discharge from the distal part of the detached GSV at the SFJ, which was also demonstrated by ultrasound during surgery. We speculate that the pressure of the deep veins or the reflux of PVs of the patients led to the backflow of sclerosing agent injected into the VVs without reaching the deep veins. Consequently, HL of the GSV may help prevent the foam sclerosing agent from flowing back into the deep vein through the GSV, which supports the need for HL of the GSV and the safety of N-UGFS. In the future, the method of direct injection of foam sclerosing agent needs to be verified with a larger sample and longer follow-up.

Although combined surgery with CT had a shorter ulcer healing time and lower ulcer recurrence rate after the intervention, the combined operation still had corresponding complications. DVT occurred in 2 limbs (1.0%); laser burn of the skin occurred in 6 limbs (3.0%); saphenous nerve injury occurred in 11 limbs (5.5%); and superficial phlebitis occurred in 15 limbs (7.5%). The common complication of CT was skin anaphylaxis to hosiery in 35 cases (9.3%). All these complications recovered through corresponding processes. We also found that the reduction in laser power on the calf reduces the chance of skin burns and saphenous nerve injury^31^. The complication of CT was the contact skin allergy of the hosiery, which might be related to the materials of the hosiery.

This study has some limitations. First, it is not a randomized controlled trial and is, therefore, more subject to bias, which may diminish the strength of our conclusions. Second, three surgical methods were performed simultaneously, which might be a good unity of intervention, but excessive intervention might exist, as some superficial veins might be unnecessarily closed. Third, the hosiery we applied might not be the same brand. Patients bought the hosiery according to a hosiery manual, which might affect the consistency of compression therapy. The reason was that the cost of hosiery was not within the scope of the health care reimbursement. Further study of compression therapy should be provided with the same brand of hosiery. Fourth, the follow-up time was short, and a longer follow-up time is needed.

In conclusion, in the treatment of active VLUs, combined treatments can shorten the ulcer healing time and reduce the ulcer recurrence rate compared with CT alone.

## Supplementary information

Dataset 1

Dataset 2
